# Progress in Gynæcology

**Published:** 1902-09-20

**Authors:** 


### PROGRESS IN GYNÆCOLOGY.

(*Concluded from page* 419.)

**Deciduoma Malignum.**—There is still considerable difference of opinion with regard to the origin of these tumours, the three principal theories being: 1. That they originate from the uterine connective tissue; 2. That they are the result of malignant changes in the decidual cells, and 3. The theory that they are the result of a proliferation of the epithelial layers covering the chorionic villi. H. J. Boldt[8] says that while the majority of those who have studied this subject maintain that deciduoma malignum is produced entirely by the products of conception, there are some very competent observers who have given good reasons to think that the growth may arise from the maternal structures. One case that he had himself observed rather supported this view. Blakesley and Sevestre[9] observe that it is important to remember that these tumours always occur soon after an abortion, or delivery at term, and they think that this fact is strongly in favour of the theory that they arise in decidual tissues. Ladinski[10] also points out that every reported case of deciduoma malignum, except one, has been preceded by pregnancy, and the evidence in that case was doubtful. He gives the average duration of life when the disease occurs after vesicular mole as six months; after abortion five months; and after labour at full term four months. J. Halliday Croom,[11] in a paper on this subject, says that the consensus of opinion favours the view that deciduoma malignum is an epitheliomatous new growth, the result of pregnancy, and is derived from the coverings of the chorionic villi. In 60 per cent. of all the cases the disease has followed that intense proliferation of the villous epithelium known as myxomatous degeneration of the placenta, or hydatid mole. Hitschmann[12] reports an instructive case occurring in a multipara, age 38 years. She missed one period, and soon after began to suffer from uterine hæmorrhage, which continued for three months. On examination the uterus was found to be enlarged, and a tumour presented at the os internum. Microscopic examination of fragments of this growth showed it to be deciduoma malignum. The uterus was extirpated, but, in spite of this, the patient died three weeks later from metastases in the thoracic and abdominal viscera, as well as in the brain.

**Primary Tuberculosis of the Cervix.**—A. H. N. Lewers[13] reports a very instructive case of primary tuberculosis of the cervix simulating cancer. The patient was a married woman, 36 years of age, who had never been pregnant. For nine months before she came under observation, there had been a vaginal discharge which was blood stained and slightly offensive. On examination, the vaginal portion of the cervix appeared to be the seat of "a growth," which was friable, bled readily, and in many respects resembled cancer. The uterus was freely movable. Believing the case to be one of malignant disease of the cervix, suitable for radical operation, Dr. Lewers performed vaginal hysterectomy. On microscopic examination, it was found that there was no evidence of cancer, but that the structure was tuberculous. The patient made a good recovery, and was in good health five years after the operation. A somewhat similar case is reported by Beyea.[14] The patient was 23 years of age, and had been married five months. Menstruation had always been irregular, and very slight in amount. For three years she had suffered from an extremely offensive and profuse leucorrhœal discharge. On vaginal examination it was found that the mucous membrane of the portio vaginalis for a distance of from 1 cm. to 2 cm. surrounding the external os was eroded, and of a bright rose-red colour. The whole surface of the cervix was hardened, indurated, and bled readily, in many respects resembling the early stage of a cauliflower epithelioma of the portio vaginalis. Within the cervical canal and dilating it was a small irregular papillary growth the size of a nut. The uterus was retroverted, and there was evidence of disease of the tubes and ovaries. The lesions were considered to be due to one of three diseases—tuberculosis of the cervix, malignant adenoma of the cervix, or syphilis of the cervix. Microscopic examination proved the disease to be tuberculous. H. Handfield-Jones[15] reports a case which had come under his observation, in which the patient, a multipara, age 30 years, had been suffering for some months from menorrhagia, and in the intervals from a foul vaginal discharge. Examination showed that the cervix was deeply excavated by tuberculous ulceration, and that the disease had extended to the upper part of the vagina.

The patient was also suffering from strumous dactylitis. As she would not submit to any radical operation, the ulcerated cervix was thoroughly scraped, and Paquelin's cautery freely applied to the base. Cicatrisation followed, and two years later the patient was found to be free from any further spread of tubercular disease in the genital tract.

**Supra-vaginal Hysterectomy for Fibroids.**—G. Crewdson Thomas[16] reports the after histories of 100 cases of supra-vaginal hysterectomy for fibroids, and from them draws the following conclusions:—(1) The operation from the patient's standpoint is an eminently satisfactory one. (2) That one ovary should be retained, but that the importance of this diminishes in proportion as the age of the patient approaches more closely the climacteric period. (3) The operation *per se* causes no tendency to insanity, but a certain number of patients complain of a diminution in their power of memory. (4) The functions of the bowel and bladder are not usually interfered with, and the sexual sensations, as far as can be gathered, are not generally influenced by the operation.

**Pelvic Inflammation.**—Bedford Fenwick[17] strongly advocates the local application of Ichthyol in the treatment of pelvic inflammation, and reports a number of cases in which excellent results were obtained. His method of application is as follows:—Fergusson's speculum is passed, the vaginal vault swabbed out with absorbent wool, and an Ichthyol pessary passed into the posterior vaginal cul-de-sac. A wool plug with string attached is then inserted in order to keep the preparation in close contact with the vaginal surface. The plug is removed in the evening, a hot antiseptic douche given, and another plug introduced. This treatment is repeated daily for a fortnight or three weeks, at the end of which time there is usually a very marked improvement in the patient's condition.

**Primary Sarcoma of the Vagina.**—H. Macnaughton-Jones[18] records an interesting case of sarcoma of the vagina. The patient, age 44 years, had suffered from a constant vaginal discharge for seven months, during which time she had run down in her general health, and had lost weight. She had been married 23 years, having had one child and no miscarriage. On examination a considerable mass was found, filling the lower part of the vagina. The growth bled very freely, and was attached by a broad base to the anterior wall of the vagina; it did not involve the external genitalia, and the inguinal glands were quite free. The growth was completely removed together with a portion of healthy mucous membrane, and no recurrence had taken place four months after the operation. On microscopic examination the growth was found to be a spindle-celled sarcoma.

8 Med. Rec., Feb. 15, 1902. 9 Lancet, July 5, 1902. 10 Med. Rec., Feb. 15, 1902. 11 Brit. Med. Jour., April 26, 1902. 12 Amer. Jour. of Med. Sci., March, 1902. 13 Lancet, April 12, 1902. 14 Amer. Jour. of Med. Sci., Nov., 1901. 15 Lancet, April 12, 1902. 16 Ibid., Feb. 1, 1902. 17 Med. Times and Hosp. Gaz., June 28, 1902. 18 Lancet, Feb. 15, 1902.

